# The Impact of Total Hip Arthroplasty Surgical Approach on Short-Term Postoperative and Patient-Reported Outcomes

**DOI:** 10.7759/cureus.45456

**Published:** 2023-09-18

**Authors:** Laura A Stock, Andrea H Johnson, Jane C Brennan, James MacDonald, Justin J Turcotte, Paul J King

**Affiliations:** 1 Orthopedic Research, Anne Arundel Medical Center, Annapolis, USA; 2 Orthopedics, Anne Arundel Medical Center, Annapolis, USA; 3 Orthopedic Surgery, Anne Arundel Medical Center, Annapolis, USA

**Keywords:** postoperative outcomes, clinically significant improvement, posterior approach, anterior approach, total hip arthroplasty (tha), patient-reported outcome measures

## Abstract

Background

While multiple studies have compared outcomes between the direct anterior approach (DAA) and posterolateral (PL) total hip arthroplasty (THA), the debate over the optimal approach remains. Proponents of the DAA suggest that its muscle-sparing properties and unrestricted rehabilitation facilitate a more rapid return to function. The majority of studies demonstrate that patient-reported outcomes (PROs) are similar between approaches beyond the one-year timeframe, but results are mixed when evaluating earlier time points. The purpose of this study was to compare clinical outcomes and PROs between DAA and PL THAs at six weeks postoperatively.

Methods

A retrospective review of 749 primary THAs (151 PL, 598 DAA) from March 2020 to November 2022 was performed. All surgeries were performed by one of the five board-certified and fellowship-trained orthopedic surgeons. All patients completed Patient Reported Outcomes Measurement Information System-Physical Function (PROMIS-PF) questionnaires preoperatively and at six weeks postoperatively. A univariate comparison of clinical outcomes (length of stay (LOS), home discharge rate, 90-day ED returns, and readmissions) and six-week PROMIS-PF scores between approaches was performed. Multivariate analysis was performed to evaluate the effect of the approach on outcomes after adjusting for baseline differences between groups.

Results

Patients undergoing DAA THA experienced significantly shorter average LOS (0.71 vs. 1.36 days, p<0.001), higher rates of home discharge (99.0 vs. 92.1%, p<0.001), and lower rates of 90-day readmissions (0.7 vs. 6.0%, p<0.001) than those undergoing the PL approach. At six weeks postoperatively, DAA patients achieved higher average PROMIS-PF scores (42.2 vs. 39.9, p=0.001). After adjusting for the Charlson Comorbidity Index and baseline physical function, the DAA was significantly associated with shorter LOS (β=-0.52, p<0.001), increased odds of home discharge (OR=5.70, p=0.001), reduced risk of 90-day readmission (OR=0.14, p=0.001), and higher PROMIS-PF scores at six weeks postoperatively (β=1.37, p=0.045).

Conclusion

In comparison to patients undergoing PL THA, those treated using the DAA experienced improved clinical and PROs over the six-week postoperative period. While both approaches resulted in satisfactory outcomes, these results support the assertion that DAA THA may result in more rapid recovery and return to function.

## Introduction

The number of total hip arthroplasty (THA) procedures performed in the United States continues to increase, with annual volume expected to top 600,000 procedures by 2030 [1]. With these increases in THA utilization, there have been great strides in improving surgical techniques, implant technology, and patient outcomes [2]. However, there is still much debate among experts on the best surgical approach for THA [3]. The adaption to more value-based care systems has helped encourage the use of newer and more patient-outcome-driven surgical approaches such as the direct anterior approach (DAA) [3-5]. The DAA has been shown to generate shorter hospital length of stay (LOS), less postoperative pain, faster functional improvement, and reduced risk of dislocation, in part due to its muscle-sparing technique [3,5,6]. Additionally, the DAA approach uses intraoperative fluoroscopic imaging to ensure the proper placement of components during surgery [5]. The posterolateral (PL) approach is one of the most common techniques used globally [3-5,7]. This approach allows for better visualization and access to the acetabulum and femur while also sparring the abductor mechanism [4,7]. The PL approach has also been linked to shorter operative times [4].

Patient-reported outcome measures (PROMs) are being highly utilized in many clinical areas, including orthopedics, in order to improve patient-centered care and clinical decision-making [8]. The Patient Reported Outcomes Measurement Information System (PROMIS) was developed in 2004 as a cohesive measurement tool that overcomes the limitations of condition-specific PROMs, including the narrow scope and administrative burden [9,10]. When evaluating longer-term PROMs in patients undergoing DAA or PL THA, patients report equivalent results between surgical approaches at anywhere from one to five years postoperatively [2,11,12]. In the shorter term, the first six to 12 weeks following THA, the differences in functional and PROs are more mixed, with some studies indicating that patients undergoing DAA THA have improved outcomes [3,13]. The purpose of this study was to compare clinical outcomes and PROMs between DAA and PL THAs at six weeks postoperatively.

## Materials and methods

This study was conducted at Anne Arundel Medical Center in Annapolis, Maryland, USA, and was deemed institutional review board exempt by the institutional clinical research committee. A retrospective review of 749 patients undergoing THA from March 6, 2020, to November 6, 2022, was performed. All patients completed a preoperative and six-week postoperative Patient Reported Outcomes Measurement Information System-Physical Function (PROMIS-PF) survey. Patient demographics, comorbidities, and postoperative outcomes were collected via electronic medical records.

Study population

All patients underwent primary THA between March 6, 2020, and November 6, 2022. All patients had to have completed a PROMIS-PF short form preoperatively and within six weeks postoperatively to be included in the study. All patients underwent either the anterior approach or PL approach THA; the decision to proceed with a particular surgical approach was made by the operating surgeon in consultation with the patient. A total of 749 patients met the inclusion criteria.

Study outcomes

The postoperative outcomes of interest included LOS, home discharge, 90-day ED return, 90-day readmission, and six-week PROMIS-PF score. The PROMIS is a network of tools designed to help clinicians measure various aspects of patient health such as pain, physical function, and mental health [14]. PROMIS-PF helps quantify functional recovery through patients’ assessments of how much difficulty they have in completing everyday tasks.

Statistical analysis

Patients were grouped by whether they had a direct anterior or posterior surgical approach. Univariate analysis including chi-square tests and two-sided independent samples t-tests were used to determine differences in patient demographics, comorbidities, and postoperative outcomes between those who achieved the minimum clinically important difference on PROMIS-PF and those who did not. A multivariate logistic regression model was generated to assess the association between the DAA and postoperative outcomes after controlling for the Charlson Comorbidity Index (CCI) and baseline PROMIS-PF score. All statistical analyses were performed using RStudio (version 4.2.2 © 2009-2023 RStudio, PBC). Statistical significance was assessed at p<0.05.

Source of funding

This study did not receive any funding.

## Results

Of the 749 patients, 598 (79.8%) patients had a direct anterior surgical approach for their THA. Patients with a DAA had a lower BMI, on average (28.79 vs. 30.76 kg/m2; p<0.001), and fewer patients with an American Society of Anesthesiologists (ASA) score over 3 (32.1% vs. 51.7%; p<0.001). Patients with a DAA had a lower overall CCI score (2.37 vs. 2.62; p=0.048) as well as fewer patients 80 years or older (6.7% vs. 13.9%; p=0.006) and fewer with renal disease (3.0% vs. 7.3%; p=0.028). Additionally, patients with a DAA had a higher baseline PROMIS-PF score than those who had a posterior approach (36.51 vs. 34.04; p<0.001) (Table 1).

**Table 1:** Patient demographics and comorbidities p-values <0.05 in bold; data are expressed as mean ± SD or n (%); CCI: Charlson Comorbidity Index; COPD: chronic obstructive pulmonary disease; AIDS: acquired immunodeficiency virus; HIV: human immunodeficiency virus; ASA: American Society of Anesthesiologists; PROMIS-PF: Patient Reported Outcomes Measurement Information System-Physical Function

Patient demographics and comorbidities	Posterior (n=151)	Anterior (n=598)	p-value
Age	67.14 ± 11.39	65.39 ± 10.37	0.087
Body mass index	30.76 ± 5.26	28.79 ± 5.13	<0.001
Sex			0.828
Female	84 (55.6)	341 (57.0)	
Male	67 (44.4)	257 (43.0)	
Non-White race	21 (13.9)	87 (14.5)	0.944
ASA 3+	78 (51.7)	192 (32.1)	<0.001
CCI score	2.62 ± 1.45	2.37 ± 1.22	0.048
Age <50	9 (6.0)	33 (5.5)	0.989
Age 50-59	30 (19.9)	123 (20.6)	0.938
Age 60-69	45 (29.8)	231 (38.6)	0.055
Age 70-79	46 (30.5)	171 (28.6)	0.725
Age 80+	21 (13.9)	40 (6.7)	0.006
Myocardial infarction	0 (0)	0 (0)	1
Congestive heart failure	0 (0)	1 (0.2)	1
Peripheral vascular disease	3 (2.0)	10 (1.7)	1
Cerebrovascular disease	1 (0.7)	18 (3.0)	0.177
Dementia	0 (0)	3 (0.5)	0.879
COPD	8 (5.3)	19 (3.2)	0.315
Rheumatoid arthritis	3 (2.0)	20 (3.3)	0.548
Peptic ulcer disease	0 (0)	0 (0)	1
Mild liver disease	1 (0.7)	1 (0.2)	0.864
Diabetes w/o chronic	16 (10.6)	42 (7.0)	0.195
Diabetes w/ chronic	0 (0)	1 (0.2)	1
Hemiplegia/paraplegia	0 (0)	0 (0)	1
Renal disease	11 (7.3)	18 (3.0)	0.028
Malignancy	0 (0)	0 (0)	1
Moderate/severe liver disease	0 (0)	0 (0)	1
Metastatic solid tumor	0 (0)	0 (0)	1
AIDS or HIV	0 (0)	1 (0.2)	1
Baseline PROMIS-PF score	34.04 ± 6.40	36.51 ± 6.25	<0.001

Postoperatively, DAA patients had a short LOS (0.71 vs. 1.36 days; p<0.001), more patients were discharged home (99.0% vs. 92.1%; p<0.001), and fewer patients had 90-day readmission (0.7% vs. 6.0%; p<0.001). Additionally, DAA patients had a higher PROMIS-PF score on average at six weeks (42.21 vs. 39.90; p=0.001). There was no difference in 90-day ED returns between approaches (Table 2).

**Table 2:** Postoperative outcomes p-values <0.05 in bold; data are expressed as mean ± SD or n (%); ED: emergency department; PROMIS-PF: Patient Reported Outcomes Measurement Information System-Physical Function; LOS: length of stay

Postoperative outcome	Posterior (n=151)	Anterior (n=598)	p-value
LOS (days)	1.36 ± 1.71	0.71 ± 1.40	<0.001
Home discharge	139 (92.1)	592 (99.0)	<0.001
90-day ED return	12 (7.9)	29 (4.8)	0.195
90-day readmission	9 (6.0)	4 (0.7)	<0.001
6-week PROMIS-PF score	39.90 ± 7.92	42.21 ± 7.67	0.001

After controlling for the CCI score and baseline PROMIS-PF score, the DAA was associated with a 1.37-point increase in the six-week PROMIS-PF score (β=1.37, 95% CI: 0.03 to 2.71; p=0.045) and a decreased LOS (β=-0.52, 95% CI: -0.77 to -0.26; p<0.001). Additionally, those who had a DAA were 5.70 times more likely to be discharged home (OR=5.70, 95% CI: 2.02 to 17.66; p=0.001) and 7.14 times less likely to have a 90-day readmission (OR=0.14, 95% CI: 0.04 to 0.45; p=0.001) (Table 3).

**Table 3:** Multivariate regression p-value <0.05 are in bold; controlling for CCI score and baseline PROMIS-PF; LOS: length of stay

Postoperative PROMIS-PF	Anterior β/OR	95% CI	p-value
6-week PROMIS-PF (β)	1.37	0.03 to 2.71	0.045
LOS (β)	-0.52	-0.77 to -0.26	<0.001
Home discharge	5.70	2.02 to 17.66	0.001
90-day readmission	0.14	0.04 to 0.45	0.001

## Discussion

The results of our study demonstrate that DAA patients were overall healthier and had better physical function preoperatively compared to PL approach patients. Further, DAA patients had improved postoperative outcomes and higher physical function at six weeks postoperatively. Unlike previous literature, our study also controlled for CCI score and baseline physical function to better assess the progression made postoperatively and found that DAA was associated with greater PROMIS-PF scores, greater home discharge, decreased LOS, and less risk for 90-day readmission.

Multiple studies have shown similar outcomes between DAA and PL approaches when considering longer-term outcomes [5,11,15]. In the shorter term, multiple studies have demonstrated the superiority of the DAA when considering outcomes such as LOS, dislocation risk, readmissions, and non-home discharge [6,12,13,16,17]. Christensen et al. investigated functional recovery between DAA and PL approaches within the first six weeks following THA and found the DAA group had a significantly shorter LOS and earlier discontinuation of assistive devices [6]. Another study evaluating early outcomes between DAA and PL approach in 150 THAs found that DAA was associated with shorter hospital stays and home discharge [13]. Zawadsky et al. also found less pain, significantly less use of assistive devices, and less narcotic use at six weeks, consistent with previous literature [6,13]. Barrett et al. conducted a prospective randomized study comparing the DAA and PL approach in 87 THA patients and also found similar results with DAA associated with earlier discharge, longer postoperative ambulation distance on postoperative days zero to two, and more patients walking unlimited and using stairs normally at six weeks [17]. Martusiewicz et al. noted improved pain, shorter LOS, and improved functional status persisting through five weeks postoperatively in patients undergoing DAA THA compared with patients undergoing PL THA [18]. Similar to these studies, our study demonstrated earlier improvement in physical function, shorter length of hospital stay, and greater home discharge for DAA. A meta-analysis by Miller et al. found that patients undergoing PL THA had no increased risk of complications compared with those undergoing DAA THA, although they did find that patients undergoing DAA had lower pain scores, decreased narcotic consumption, and improved hip function [3].

While patients undergoing DAA THA do appear to have improved short-term functional outcomes and fewer complications, PROMs show more inconsistent short-term results when comparing DAA and PL approaches. Martusiewicz et al. investigated early outcomes of 111 THAs weekly for up to six weeks, comparing DAA and PL approaches, and found that postoperatively, patients undergoing DAA had improved PROMIS-PF and modified Harris hip scores up to five weeks [18]. Christensen et al. investigated PROMs between DAA and PL approaches within the first six weeks following THA including modified Harris hip scores, pain and function subcomponent scores, lower extremity function scale, single assessment numeric evaluation, and SF-12 mental and physical scores and found no differences between groups at the six week follow up visit [6]. Quinzi et al. investigated 409 THA patients and compared PROMIS-PF and pain interference (PI) scores between approaches and found no significant differences in approaches for PROMIS-PF and PI score improvement at any postoperative time point (six weeks, six months, or one year), although the DAA was associated with significantly greater PROMIS-PF scores at all time points [2]. Our study did show improved PROMIS-PF in patients undergoing DAA THA at six weeks postoperatively, although both DAA and PL THA patients showed improvement from baseline to six weeks postoperatively. Studies that examine longer-term PROMs also have somewhat mixed results, although most show fewer differences in PROMs between approaches. Maldonado et al. found that patients undergoing DAA THA achieved superior quality of life outcomes at a minimum two-year follow-up, although all other PROMs had comparable scores between the two groups [12]. Barrett et al. found no differences in PROMs between patients undergoing DAA and PL THA after three months postoperatively [17].

This study does not come without limitations. First, we conducted a retrospective data review; therefore, our results may not be representative of the general population for THA. Our second limitation is selection bias between groups. Given the nature of both surgical approaches, certain patients may have been selected for a particular approach due to overall health, case complexity, or surgeon preference. However, we were able to adjust for some of these differences by controlling for CCI. The sample size was also limited due to the requirement that all included patients had to have completed both a preoperative and six-week postoperative PROMIS-PF score. Finally, due to our study objective of investigating short-term outcomes, our follow-up time was limited to only six weeks.

## Conclusions

Both approaches saw an increase in PROMIS-PF in the first six weeks and an overall low complication rate postoperatively. In comparison to patients undergoing PL THA, those treated using the DAA experienced improved clinical and PROs over the six-week postoperative period. These results support the assertion that DAA THA may result in more rapid recovery and return to function.
